# Dimethyl Fumarate Enhances Venetoclax-Induced Cell Death by Inhibiting Mitochondrial Respiration and Cell Cycle Control in Colorectal Cancer

**DOI:** 10.3390/life16081250

**Published:** 2026-07-28

**Authors:** Simon Wagner, Anne Schroeder, Sara Martina Steinmann, Claudia Kunst, Kirstin Pollinger, Manuela Gunckel, Elisabeth Aschenbrenner, Martina Müller, Karsten Gülow

**Affiliations:** 1 Department of Internal Medicine I, Gastroenterology, Hepatology, Endocrinology, Rheumatology and Infectious Diseases, University Hospital Regensburg, Franz-Josef-Strauß-Alle 11, 93053 Regensburg, Germany; sim.wagner@uke.de (S.W.); sara.steinmann@ukr.de (S.M.S.); claudia.kunst@ukr.de (C.K.); kirstin.pollinger@ukr.de (K.P.); manuela.gunckel@klinik.uni-regensburg.de (M.G.); elisabeth.aschenbrenner@ukr.de (E.A.); martina.mueller-schilling@ukr.de (M.M.); 2 Translational Oncology (TRON) at the University Medical Center of Johannes Gutenberg University, Freiligrathstraße 12, 55131 Mainz, Germany; anne.schroeder@tron-mainz.de

**Keywords:** dimethyl fumarate (DMF), Venetoclax (ABT-199), apoptosis, autophagy, metabolism, cell cycle

## Abstract

Colorectal cancer (CRC) is a leading cause of cancer death, with resistance and apoptosis evasion—often via Bcl-2—representing major challenges. The redox-modulating drug dimethyl fumarate (DMF) has demonstrated efficacy in hematologic malignancies; however, its potential in solid tumors remains unclear. Here, we show that DMF, especially in combination with the Bcl-2 inhibitor venetoclax (ABT-199), induces apoptosis in HCT-116 CRC cells. DMF impairs mitochondrial respiration, causing membrane hyperpolarization, ATP depletion, autophagy, and cell cycle arrest. Combined treatment increases metabolic stress, reduces proliferation, and induces sustained G2 arrest with downregulation of cyclins and CDKs. These findings highlight a combined effect targeting redox balance and apoptosis in CRC. Given their clinical availability, DMF and ABT-199 represent a promising combination for further preclinical evaluation.

## 1. Introduction

Colorectal cancer (CRC) remains one of the most prevalent and deadly cancers worldwide [1]. While early-stage disease can often be treated successfully [2,3], the prognosis for patients with metastatic CRC remains poor due to high recurrence rates [4,5] and the development of resistance to standard treatments such as 5-fluorouracil (5-FU)-based chemotherapy, targeted therapies (e.g., anti-EGFR or anti-VEGF antibodies), and immune checkpoint blockade [5,6,7,8,9].

To overcome therapeutic resistance and improve outcomes in metastatic CRC, the development of novel agents with alternative mechanisms of action is essential [9,10]. Dimethyl fumarate (DMF), an U.S. Food and Drug Administration (FDA)-approved drug for multiple sclerosis [11], has emerged as a promising candidate due to its ability to modulate cellular redox balance, inhibit pro-inflammatory signaling pathways such as nuclear factor kappa B (NF-κB), and induce cell death in various cancer cell types [12,13,14,15,16].

DMF is an effective NF-κB inhibitor that has demonstrated significant antitumor activity in various T-cell malignancies [9,14,15,16,17]. DMF covalently modifies free thiol groups via Michael addition, leading to monosuccinylation of target proteins and modulation of redox-sensitive signaling pathways [15]. We identified thioredoxin-1 (Trx1) as a key redox-sensitive DMF target. Monosuccinylation impairs Trx1 activity, preventing reduction in nuclear NF-κB subunits. Consequently, oxidized NF-κB shows reduced DNA-binding capacity, diminishing transcription of anti-apoptotic genes, including inhibitor of apoptosis (IAP) family members. This redox-mediated suppression of survival signaling promotes ripoptosome formation, triggering apoptosis or necroptosis in malignant T cells [14,15]. In our recent multicenter phase II clinical trial (EudraCT 2014-000924-11; NCT02546440), this mechanism was supported by clinical data, as DMF treatment demonstrated high efficacy in patients with cutaneous T-cell lymphoma (CTCL), leading to a marked reduction in tumor burden and cutaneous manifestations [18]. Importantly, we also identified that DMF inhibits metastasis in CTCL through an NF-κB-independent mechanism, suggesting that its antitumor activity extends beyond redox modulation and NF-κB signaling. This indicates a broader therapeutic potential of DMF in limiting both tumor growth and dissemination [14]. This finding suggests that the therapeutic potential of DMF may extend beyond T-cell malignancies, potentially offering broader applicability in treating solid tumors [16]. NF-κB shows constitutive activation in a substantial subset of colorectal cancers, whereas other tumor entities progress independently from NF-κB signaling [19,20]. The ability of DMF to act through both NF-κB-dependent and -independent pathways highlights its promise as a broad-spectrum anticancer agent.

In addition to its anti-tumorigenic effects, DMF shows markedly enhanced effects in combination with the Bcl-2 inhibitor venetoclax (ABT-199) in T-cell malignancies. Bcl-2 prevents mitochondrial outer membrane permeabilization and is a key survival factor upregulated in many cancers. ABT-199, a highly selective Bcl-2 inhibitor approved for relapsed or refractory chronic lymphocytic leukemia (CLL) [21,22,23] is increasingly tested in combination therapies [18,21,24,25]. Since CRC cells often evade apoptosis via Bcl-2 and related pathways, combining DMF with ABT-199 may represent a novel strategy to overcome treatment resistance in CRC.

Furthermore, the favorable safety profile and low off-target activity of DMF make it an attractive candidate for CRC treatment. Its ability to enhance efficacy while maintaining tolerability could provide a major advantage over conventional chemotherapy [18]. Investigating DMF, alone or combined with Bcl-2 inhibitors, in CRC could yield novel strategies to overcome apoptotic resistance. Further preclinical studies are warranted to clarify underlying mechanisms and assess efficacy.

Our results show that DMF induces CRC cell death, markedly enhanced by ABT-199. DMF causes adenosine triphosphate (ATP) depletion via mitochondrial hyperpolarization, leading to cell cycle arrest, suppressed proliferation, and autophagy. This combination targets both apoptotic resistance and metabolic vulnerabilities in CRC.

## 2. Materials and Methods

Chemicals. ABT-199 was from Selleck Chemicals LLC. (Houston, TX, USA); cell culture media and supplements from Gibco (Thermo Fisher Scientific, Waltham, MA, USA); fetal calf serum (FCS) from Anprotec e.K. (Bruckberg, Germany). Unless otherwise specified, chemicals were from Sigma-Aldrich (St. Louis, MO, USA).

Cells. HCT-116 cells (ATCC CCL-247) and HCT-15 (ATCC CCL-225) were cultured in McCoy’s 5A (Modified) medium (Gibco, Waltham, MA, USA) with GlutaMAX™ (Gibco) and 10% fetal calf serum (FCS; Anprotec, Bruckberg, Germany) at 37 °C in a humidified 5% CO_2_ atmosphere.

Antibodies. Primary antibodies against cyclin-dependent kinase 1 (CDK1); cyclin-dependent kinase 4 (CDK4); cyclin-dependent kinase 6 (CDK6); cyclins A2, B1, D1, E1; and microtubule-associated protein 1 light chain 3 beta (LC3B) were from Cell Signaling Technology. β-actin and horseradish peroxidase (HRP)-conjugated anti-mouse and anti-rabbit secondary antibodies were from Sigma-Aldrich.

Flow cytometric analysis. Flow cytometry was performed on a BD LSRFortessa™ Cell Analyzer (Becton, Dickinson and Company, Franklin Lakes, NJ, USA). Cell death was determined by Annexin V/4′,6-diamidino-2-phenylindole (DAPI) staining in Annexin V binding buffer (Becton Dickinson, Franklin Lakes, NJ, USA). Specific cell death (%) was calculated as:

Specific cell death (%) = (% experimental cell death − % spontaneous cell death)/(100% − % spontaneous cell death) × 100 [26].

For caspase inhibition, cells were pretreated with 50 µM Z-VAD (Selleck Chemicals, Houston, TX, USA) for 30 min before compound addition.

Mitochondrial membrane potential (ΔΨm) was measured by staining with 5 nM tetramethylrhodamine ethyl ester (TMRE; Thermo Fisher Scientific, Waltham, MA, USA) for 30 min and analysis in the phycoerythrin (PE) channel. For disruption of oxidative phosphorylation, 30 µM trifluoromethoxy carbonylcyanide phenylhydrazone (FCCP; Selleck Chemicals) was added 3 h before analysis.

Proliferation was assessed by staining with 0.5 µM Cell Proliferation Staining Reagent—Green Fluorescence (Abcam, Cambridge, UK) and overnight incubation before treatment; measurements were taken at 24, 48, and 72 h in the fluorescein isothiocyanate (FITC) channel.

For cell cycle analysis, cells were treated with ABT-199 or DMF for 24 or 48 h, fixed in 100% ethanol (Carl Roth GmbH + Co. KG, Karlsruhe, Germany) at −20 °C overnight, digested with ribonuclease A (1 mg/mL, 50 µL per sample; Sigma-Aldrich) for 20 min, and stained with DAPI (0.5 µg/mL; Becton Dickinson, Pacific Blue channel). Cell cycle distribution (G1, S, G2) was analyzed using FlowJo™ software (v10.10.1, FlowJo LLC., Ashland, OR, USA).

Western Blot. Western blotting was performed as described previously [27]. Protein lysates were separated on Mini-PROTEAN^®^ TGX™ gels (Bio-Rad Laboratories, Hercules, CA, USA), transferred to polyvinylidene difluoride (PVDF) membranes (Thermo Fisher Scientific), and developed using a ChemiDoc™ XRS+ imaging system (Bio-Rad). Densitometry was performed with Image Lab 6.1 software (Bio-Rad).

Cell Viability/ATP measurement. Cellular ATP content, as a marker of viability, was measured using the CellTiter-Glo^®^ Luminescent Cell Viability Assay (Promega Corporation, Madison, WI, USA) according to the manufacturer’s instructions.

Statistical analysis. Data were analyzed using GraphPad Prism 8.0 and are presented as mean ± standard deviation. Pairwise comparisons were performed using Student’s *t*-test or ANOVA, as appropriate. *p* < 0.05 was considered statistically significant (* *p* < 0.05, ** *p* < 0.005, *** *p* < 0.0005, **** *p* < 0.0001).

Language editing. For additional checking of spelling, punctuation, and grammar the large language model ChatGPT (Version 5.5, OpenAI, San Francisco, CA, USA) was used during manuscript preparation.

## 3. Results

### 3.1. Induction of Cell Death by DMF and ABT-199

CRC frequently displays increased NF-κB activity and upregulation of Bcl-2 family proteins, contributing to therapy resistance and evasion of apoptosis [19,20,21,24]. Since DMF targets redox-sensitive survival pathways and Bcl-2 inhibition can restore apoptotic signaling, we investigated whether combining DMF with the Bcl-2 inhibitor ABT-199 could enhance cell death in CRC cells.

**Figure 1:**
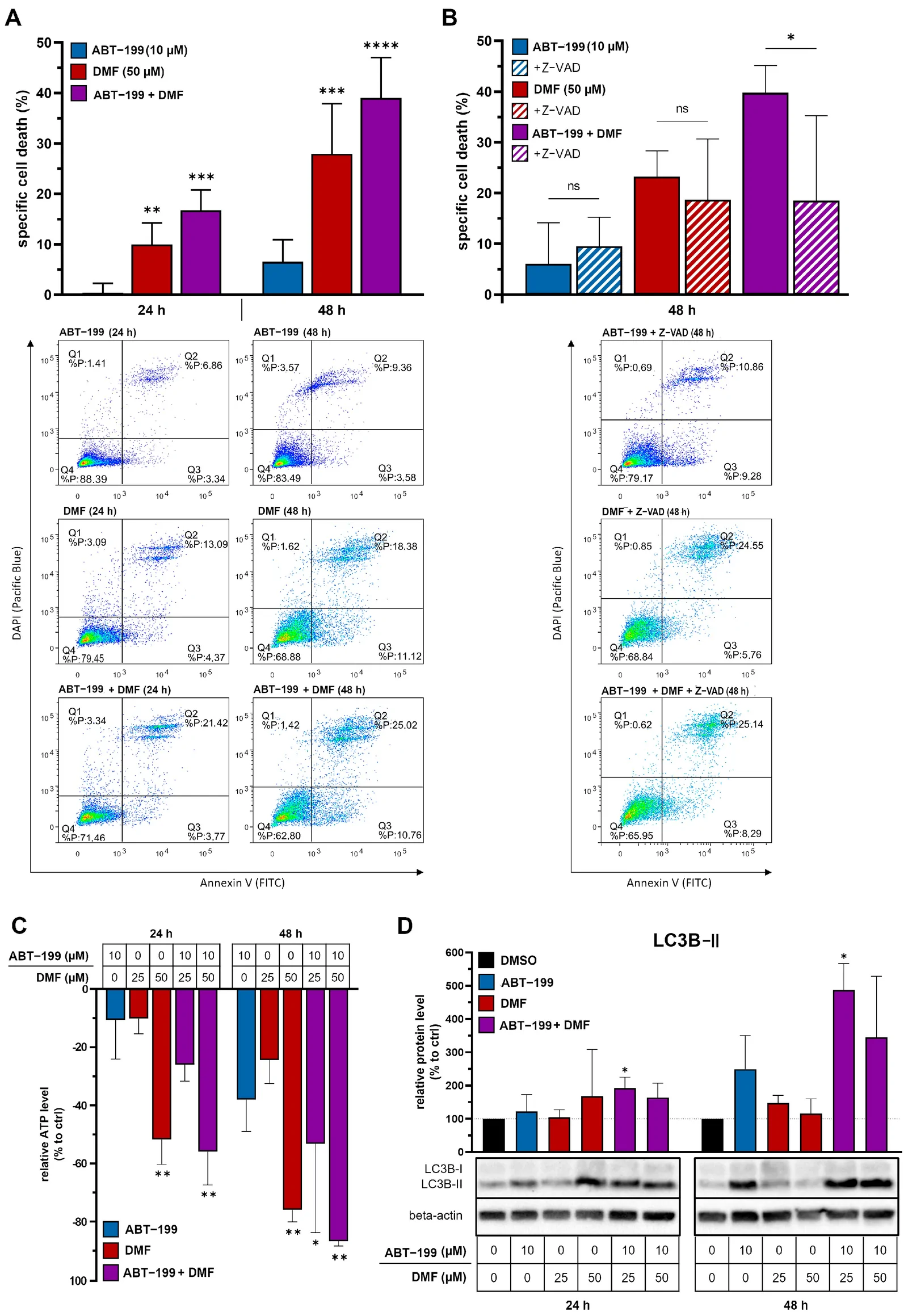
(**A**,**B**) Enhanced cytotoxicity in HCT-116 cells through combined ABT-199 and DMF treatment. Flow cytometric analysis of specific cell death by Annexin V/DAPI staining (**A**) without and (**B**) with caspase inhibition by Z-VAD (50 µM). Cells were treated with dimethyl sulfoxide (DMSO) as control, 10 µM ABT-199, 25 or 50 µM DMF or the combination of 10 µM ABT-199 with 25 or 50 µM DMF (*n* = 3). (**C**) Decrease in intracellular ATP under DMF, ABT-199 and the combination of both. Luminometric measurement of the amount of ATP in HCT-116 cells. Relative decrease in ATP amount compared to control (DMSO) in % (*n* = 2) after treatment with 10 µM ABT-199, 25 or 50 µM DMF or the combination of 10 µM ABT-199 with 25 or 50 µM DMF for 24 h and 48 h. (**D**) Induction of autophagy in HCT-116 cells by combined ABT-199 and DMF therapy. Western blot analysis of LC3B expression in HCT-116 cells treated with DMSO (control), 10 µM ABT-199, 25 or 50 µM DMF or the combination of 10 µM ABT-199 with 25 or 50 µM DMF. Densitometric quantification (*n* = 3) and representative Western blot analysis after 24 h and 48 h. * *p* < 0.05; ** *p* < 0.005; *** *p* < 0.0005; **** *p* < 0.0001; ns, *p* ≥ 0.05.

To assess cell death, HCT-116 cells were analyzed by flow cytometry. ABT-199 alone induced 0.4% specific cell death at 24 h and 6.5% at 48 h, whereas DMF alone caused 10.0% and 28.0%, respectively. The combination yielded the highest levels: 16.7% at 24 h and 39.0% at 48 h. Z-VAD, a pan-caspase inhibitor, markedly reduced combination-induced death, indicating caspase-dependent apoptosis (Figure 1A,B). Thus, DMF induces substantial CRC cell death, which is enhanced by Bcl-2 inhibition.

To evaluate whether the observed effects extend beyond HCT-116 cells, we additionally analyzed DMF and ABT-199 treatment in HCT-15 cells. HCT-15 cells were more sensitive to ABT-199, which induced more than 25% specific cell death after both 24 and 48 h, whereas DMF alone had no detectable effect after 24 h and induced only approximately 10% cell death after 48 h. The combination resulted in only a slight further increase compared with ABT-199 alone (Supplementary Figure S1A).

We next investigated additional cell death pathways underlying this effect.

### 3.2. Reduction in Cellular Energy Metabolism and Induction of Autophagy

Building on our observation that DMF, particularly in combination with ABT-199, induces pronounced cell death in CRC cells (Figure 1A,B), we next examined whether this effect is associated with alterations in cellular metabolism and induction of stress responses.

Dimethyl fumarate is a methyl ester of fumarate, an intermediate of the Krebs cycle. Due to this structural similarity, DMF can interfere with metabolic processes, particularly by limiting the availability of reducing equivalents such as reduced nicotinamide adenine dinucleotide (NADH). Previous studies have demonstrated that DMF diminishes mitochondrial respiration [28], which may contribute to metabolic stress. In addition, ABT-199 has been reported to destabilize the mitochondrial membrane potential and impair oxidative phosphorylation [29]. We therefore investigated whether DMF and its combination with ABT-199 affect cellular energy metabolism in CRC cells.

ATP levels were measured using a luminescence-based viability assay. Both DMF and ABT-199 reduced ATP in a dose- and time-dependent manner. At 24 h, 10 µM ABT-199 or 25 µM DMF alone caused ~10% reduction, whereas the combination decreased ATP by ~25%. The strongest effect (10 µM ABT-199 + 50 µM DMF) reduced ATP by ~56% at 24 h and ~87% at 48 h (Figure 1C).

Comparable ATP depletion was also observed in HCT-15 cells. At 24 h, ABT-199 or DMF alone reduced intracellular ATP levels by more than 20%, whereas the combination caused a reduction of up to approximately 60%. After 48 h, ATP levels were reduced by more than 30% following ABT-199 treatment, by more than 50% following DMF treatment, and by more than 60% following combined treatment (Supplementary Figure S1B). Thus, despite the differences in cell death induction, both colorectal cancer cell lines displayed pronounced treatment-associated ATP depletion. As the primary aim of this study was to characterize the underlying mechanisms of DMF/ABT-199 treatment, subsequent in-depth mechanistic analyses focused on the well-established HCT-116 model.

The pronounced ATP depletion observed after DMF and ABT-199 treatment may trigger compensatory stress responses, including autophagy.

Autophagy degrades and recycles damaged cellular components, with LC3B-II serving as a key marker through its association with autophagosome membranes. As ATP depletion can trigger autophagy, we analyzed LC3B-II by Western blot. DMF/ABT-199 (10 µM/25 or 50 µM) markedly increased LC3B-II at 24 and 48 h. DMF alone caused a transient rise at 24 h, whereas ABT-199 alone showed only a weak LC3B-II increase after 24 h but a more pronounced effect after 48 h. This delayed response may reflect a secondary autophagic stress reaction caused by prolonged Bcl-2 inhibition and progressive mitochondrial/metabolic impairment rather than an immediate autophagy-inducing effect. In contrast, the combination induced a robust, sustained LC3B-II elevation, consistent with an autophagy-associated response under combined metabolic stress, and exceeded the effects of either single treatment, particularly at the earlier 24 h time point (Figure 1D).

Together, these results show that DMF/ABT-199 impairs energy metabolism and promotes LC3B-II accumulation consistent with an autophagy-associated stress response in CRC cells. To investigate the mechanisms of ATP depletion, we next examined mitochondrial membrane potential and function.

### 3.3. Hyperpolarization of the Mitochondrial Membrane Potential by DMF

Given that DMF and ABT-199 markedly reduced ATP levels and induced autophagy (Figure 1C,D), we next sought to determine the underlying mitochondrial mechanisms contributing to this energy deficit.

ATP production in mitochondria depends on the respiratory chain, which establishes a proton gradient across the inner mitochondrial membrane, generating the mitochondrial membrane potential (∆Ψm). Changes in ∆Ψm reflect alterations in mitochondrial function. To assess this, HCT-116 cells were stained with tetramethylrhodamine ethyl ester (TMRE), and ∆Ψm was analyzed by flow cytometry.

**Figure 2:**
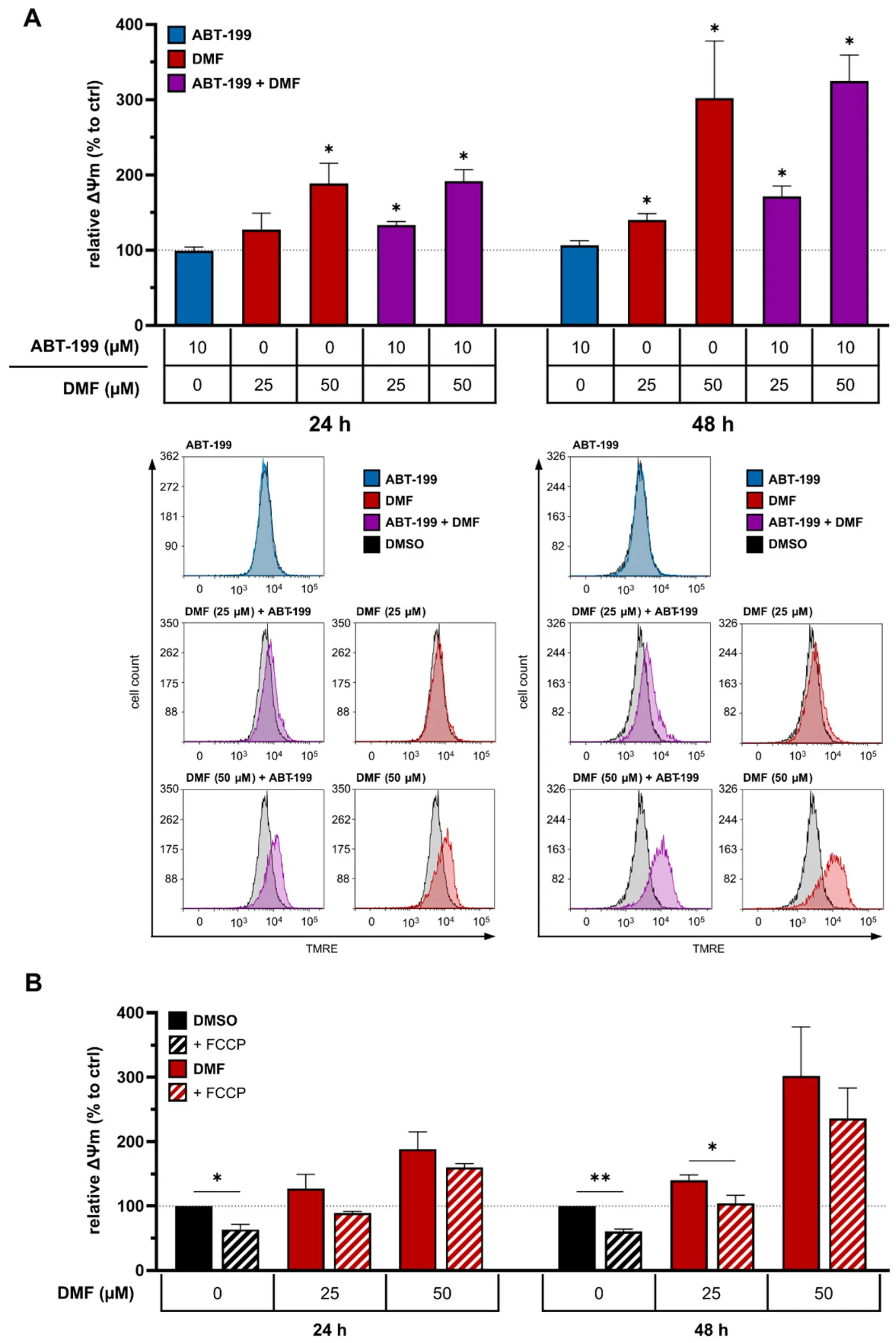
(**A**) DMF-induced hyperpolarization of the mitochondrial membrane potential (ΔΨm). Flow cytometric analysis of ΔΨm in HCT-116 cells and relative changes in % (*n* = 3) after treatment with 0.15% DMSO as control, 10 µM ABT-199, 25 or 50 µM DMF or the combination of 10 µM ABT-199 with 25 or 50 µM DMF (as indicated) for 24 h and 48 h. Quantification of flow cytometric data (upper panel) and histograms (lower panel). (**B**) Reduction in DMF-induced hyperpolarization of ∆Ψm by the mitochondrial uncoupler FCCP. Data are depicted as relative ΔΨm in % (*n* = 3) after treatment with 0.15% DMSO (control), 25 or 50 µM DMF in the presence or absence of the mitochondrial uncoupler FCCP (30 µM) for 24 h and 48 h, respectively. * *p* < 0.05; ** *p* < 0.005.

Treatment with DMF led to a significant, dose- and time-dependent hyperpolarization of ∆Ψm. This effect was observed both with DMF monotherapy and in combination with ABT-199, with 50 µM DMF inducing a stronger hyperpolarization than 25 µM. The effect was also more pronounced at 48 h compared to 24 h. In contrast, ABT-199 alone had no detectable impact on ∆Ψm (Figure 2A).

We hypothesized that DMF impairs cellular respiration by blocking electron flow through the electron transport chain [30]. This would lead to proton accumulation in the intermembrane space and cause ∆Ψm hyperpolarization, thereby reducing the efficiency of ATP production. To evaluate this possible mechanism, we used FCCP, a mitochondrial uncoupler that dissipates ∆Ψm by allowing protons to bypass ATP synthase. As expected, FCCP treatment markedly reduced ∆Ψm in untreated cells and reversed the DMF-induced hyperpolarization in treated cells (Figure 2A,B). These findings support the notion that DMF disrupts mitochondrial energy production by interfering with electron transport.

In summary, DMF induces a pronounced hyperpolarization of the mitochondrial membrane potential, which is consistent with reduced ATP production and metabolic stress. Since energy availability directly affects cellular proliferation and cell cycle progression, we next examined the impact of DMF and ABT-199 on these processes in CRC cells.

### 3.4. Inhibition of Cell Proliferation Through Cell Cycle Arrest

As ATP depletion can impair proliferation, we next analyzed how DMF and ABT-199 influence cell cycle dynamics and cellular proliferation.

ATP is essential for cell proliferation, as it provides the energy required for DNA synthesis and cell division. To assess proliferation, HCT-116 cells were stained with a cell proliferation dye prior to treatment, and dye dilution was measured by flow cytometry at 24, 48, and 72 h, using the 24 h value as baseline.

**Figure 3:**
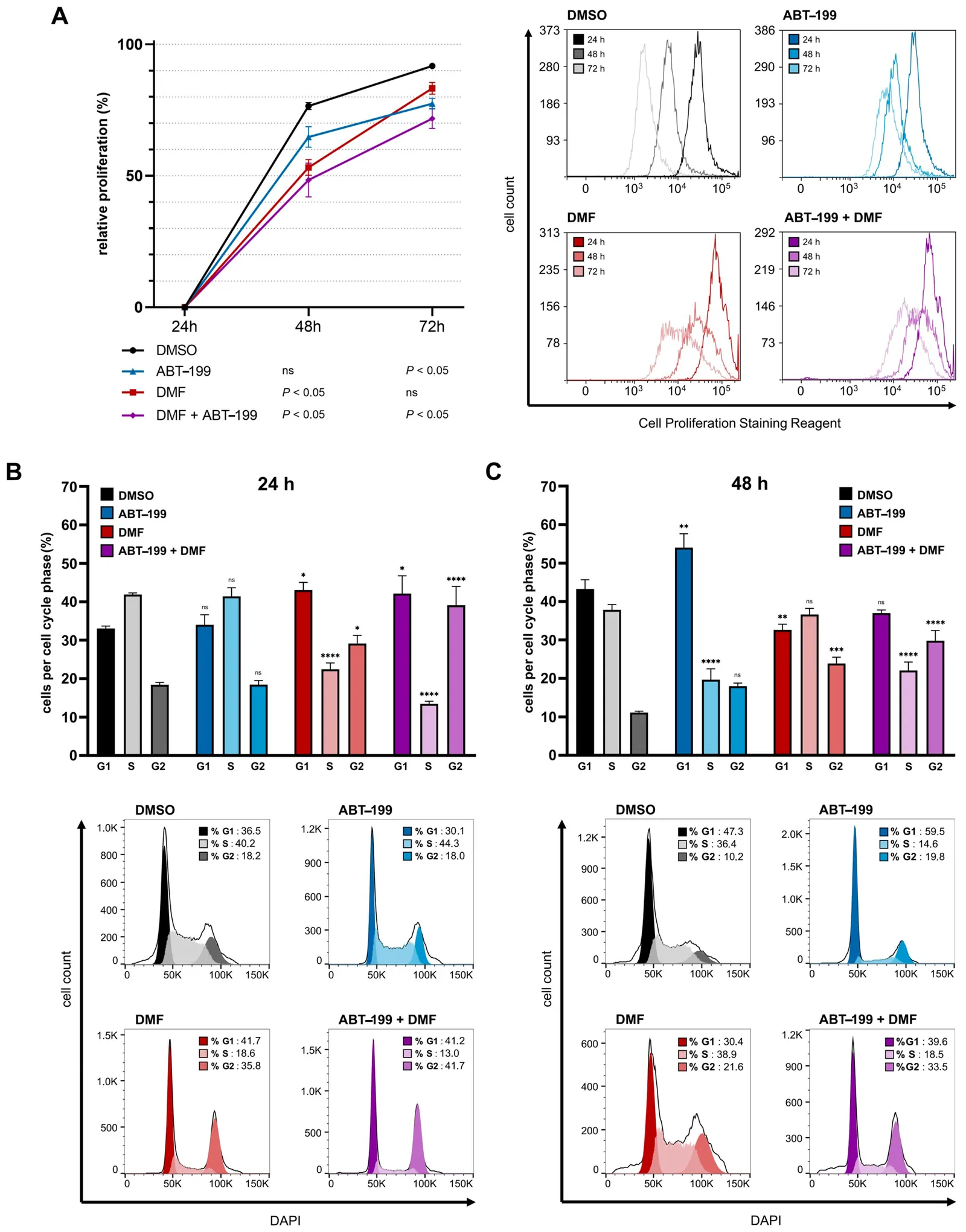
(**A**) Inhibition of cell proliferation by ABT-199, DMF and their combination. Flow cytometric analysis of cell proliferation in HCT-116 cells after treatment with 0.3% DMSO (control), 10 µM ABT-199, 50 µM DMF or the combination of both agents for up to 72 h. Relative cell proliferation in % of the measured value after 24 h (*n* = 3, left) and representative measurements (right). (**B**,**C**) Cell cycle arrest induced by ABT-199, DMF and their combination. Flow cytometric analysis of cell cycle distribution in HCT-116 cells after treatment with 0.15% DMSO (control), 10 µM ABT-199, 50 µM DMF or the combination of both agents. Quantification of cells in each cell cycle phase (*n* = 3, upper panel) and representative measurements (lower panel) after (**B**) 24 h and (**C**) 48 h. * *p* < 0.05; ** *p* < 0.005; *** *p* < 0.0005; **** *p* < 0.0001; ns, *p* ≥ 0.05.

All treatments reduced cell proliferation to varying degrees. ABT-199 alone decreased proliferation by 11.8% at 48 h and 14.4% at 72 h. DMF exhibited a more immediate effect, reducing proliferation by 23.3% at 48 h, although this effect declined to 8.5% at 72 h. The combination of DMF and ABT-199 produced the strongest inhibition, reducing proliferation by 28.2% at 48 h and 20.0% at 72 h (Figure 3A).

To elucidate the underlying mechanisms of this reduction, we analyzed cell cycle distribution after treatment. DNA was stained with DAPI, and cell cycle profiles were examined by flow cytometry at 24 and 48 h. Cell cycle arrest was observed across all treatment conditions, though with distinct profiles.

DMF and the combination therapy with ABT-199 induced a significant G1 and G2 phase arrest accompanied by a reduction in S phase at 24 h, with the combination showing a particularly strong G2 arrest. At 48 h, the cell cycle profile of DMF-treated cells normalized except for a persistent G2 arrest. In contrast, the combination therapy maintained a pronounced G2 arrest and a continued reduction in the S phase. ABT-199 alone primarily caused G1 arrest and a reduction in S phase entry (Figure 3B,C).

Taken together, these findings indicate that reduced proliferation is associated with treatment-induced cell cycle arrest. The combination of DMF and ABT-199 elicited the most robust and sustained effects on both proliferation and cell cycle progression, whereas ABT-199 showed a delayed impact and DMF a transient one.

### 3.5. Changes in Cellular Cyclin and CDK Levels

**Figure 4:**
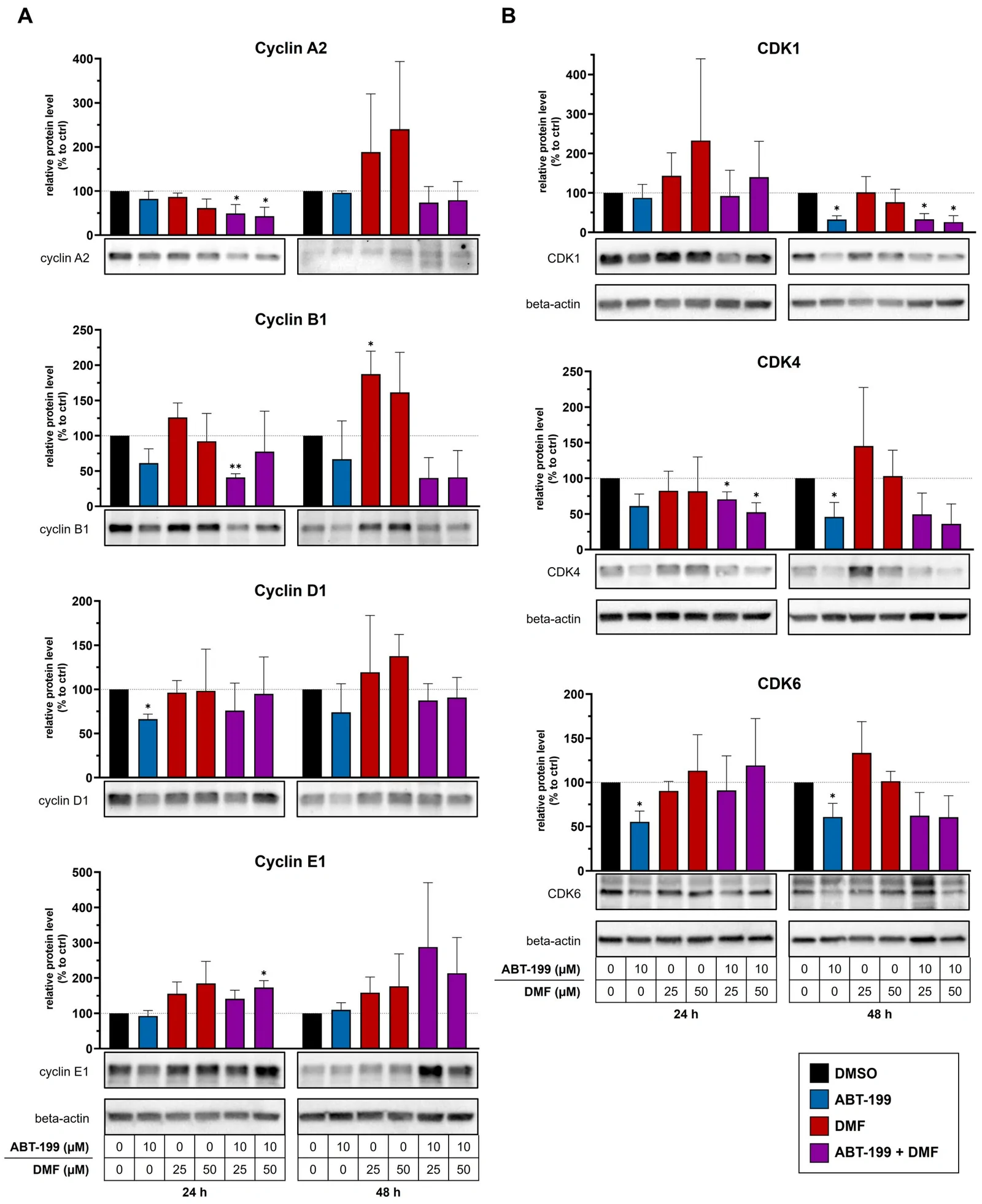
Effects of DMF, ABT-199 and the combination of both agents on cellular cyclin and CDK levels. Western blot analysis of HCT-116 cells treated with 0.3% DMSO (control), 10 µM ABT-199, 25 or 50 µM DMF or the combination of 10 µM ABT-199 with 25 or 50 µM DMF for 24 h and 48 h. Densitometric quantification (*n* = 3) and representative Western blots for (**A**) cyclin A2, cyclin B1, cyclin D1, cyclin E1 and (**B**) CDK1, CDK4 and CDK6 after 24 h and 48 h. For panel A, the same membrane was sequentially probed for the indicated cyclins after stripping and reprobing; therefore, one shared beta-actin loading control is shown for the corresponding cyclin blots. * *p* < 0.05; ** *p* < 0.005 compared to DMSO.

Since the observed proliferation defects were accompanied by distinct cell cycle arrests, we next examined whether these effects were reflected by changes in key regulators of cell cycle progression. To this end, protein levels of selected cyclins and cyclin-dependent kinases (CDKs) were analyzed by Western blot at 24 and 48 h post-treatment (Figure 4).

At 24 h, cyclin A2 levels were decreased across all treatment conditions, with the most pronounced reduction seen in cells treated with the combination of 10 µM ABT-199 and either 25 or 50 µM DMF. Cyclin B1 levels were similarly reduced by the combination treatment, whereas DMF alone had only a minor effect. Cyclin D1 was significantly downregulated following ABT-199 monotherapy, while little change was observed under other conditions. In contrast, cyclin E1 levels increased in response to DMF at both concentrations and under combination treatment, with the strongest induction seen at 50 µM DMF (Figure 4A).

At 48 h, cyclin A2 expression was elevated in cells treated with DMF alone but remained low in the combination groups. Cyclin B1 remained suppressed in the combination group, while cyclin D1 and E1 showed only minor changes across all treatments (Figure 4A).

The observed cyclin expression patterns were consistent with the cell cycle profiles determined by flow cytometry. The reduction in cyclin A2 and cyclin B1, particularly under combined treatment, is compatible with impaired progression through the G2/M transition. Likewise, the reduction in cyclin D1 following ABT-199 treatment is consistent with the observed accumulation of cells in G1. The increase in cyclin E1 after DMF treatment may reflect a compensatory response to impaired cell cycle progression rather than directly indicating blockade of a specific transition. Notably, the partial normalization of the cell cycle profile under DMF monotherapy at 48 h coincided with partial recovery of cyclin expression, which may be related to the short half-life of DMF.

To further support these findings, CDK protein levels were analyzed. ABT-199 reduced CDK1, CDK4, and CDK6 levels at both time points. The combination of ABT-199 with 25 µM DMF led to a decrease in CDK1, while 50 µM DMF lowered CDK4 levels at 24 h. At 48 h, all CDKs were suppressed under combination treatment. In contrast, DMF monotherapy did not significantly alter CDK1, CDK4, or CDK6 protein levels at either 24 or 48 h (Figure 4B).

These findings suggest that changes in CDK abundance may contribute to the observed alterations in cell cycle distribution. Reduced CDK4 and CDK6 levels are consistent with impaired G1 progression, whereas reduced CDK1 expression may contribute to impaired progression through the G2/M checkpoint. However, cell cycle transitions are additionally regulated by CDK activity, phosphorylation status, checkpoint signaling, and cyclin–CDK complex formation, which were not directly assessed in the present study.

Taken together, DMF and ABT-199 exert an overadditive effect in CRC by engaging multiple mechanisms that suppress tumor growth. The combination induces apoptosis, triggers an energetic crisis that activates autophagy, and slows proliferation. Persistent cell cycle arrest, driven by coordinated cyclin/CDK modulation and impaired mitochondrial respiration with ATP depletion, further limits tumor expansion.

Given their clinical availability and favorable safety profiles [11,18,21,23], DMF and ABT-199 represent a promising strategy to overcome resistance in colorectal cancer. These findings support further preclinical and translational studies on repurposing DMF for solid tumors, particularly in combination with targeted apoptosis modulators.

## 4. Discussion

CRC remains a major clinical challenge due to high incidence, frequent therapy resistance, and poor prognosis in advanced stages [1,3]. In 2024, it is projected to be the second leading cause of cancer death and the third most diagnosed cancer in the United States, with ~152,810 new cases and 53,010 deaths [3]. A hallmark of CRC is apoptosis evasion through activation of pro-survival pathways such as NF-κB and upregulation of anti-apoptotic Bcl-2 family members [31,32,33]. NF-κB activation, common in CRC, drives tumor progression and therapy resistance [34], while Bcl-2 overexpression further enhances chemoresistance [33]. While targeted therapies and immune checkpoint inhibitors benefit some patients with mismatch repair-deficient (MSI-H) CRC, durable responses are rare in microsatellite-stable (MSS) tumors, which comprise the majority of cases. This underscores the urgent need for effective treatments for MSS CRC [35].

To address these limitations, we tested a dual-targeting strategy combining DMF, a redox modulator that inhibits NF-κB, with the Bcl-2 inhibitor ABT-199. DMF induced dose- and time-dependent CRC cell death, markedly enhanced by ABT-199.

Beyond apoptosis, DMF profoundly altered cellular metabolism. DMF, especially with ABT-199, caused severe ATP depletion (up to 87% within 48 h) and mitochondrial hyperpolarization. These findings align with pancreatic cancer studies showing DMF suppresses mitochondrial respiration and glycolysis, reducing oxygen consumption and ATP production by up to 70% [29]. Mitochondrial membrane hyperpolarization further indicates electron transport chain dysfunction.

Similarly, ABT-199 impairs mitochondrial bioenergetics independently of apoptosis. In acute myeloid leukemia and breast cancer models, it reduces oxygen consumption by inhibiting mitochondrial respiration and disrupting the tricarboxylic acid cycle, indicating a direct metabolic effect [29]. Moreover, surviving acute myeloid leukemia cells can maintain mitochondrial membrane potential via ATP-dependent compensatory mechanisms [36]. These findings indicate that DMF and ABT-199 cooperatively impair mitochondrial function, disrupting energy production and reinforcing metabolic stress in CRC cells [37].

The energetic crisis induced by DMF/ABT-199 was accompanied by sustained LC3B-II accumulation, supporting an autophagy-associated stress response rather than formally proving functional autophagy induction. While DMF alone caused only a transient increase, co-treatment elicited a robust, prolonged response, likely due to the combined metabolic and mitochondrial stress imposed by both compounds. In CRC, autophagy and its associated processes are known to play dual roles, either supporting cell survival under stress or promoting cell death, depending on the context [38,39]. Xie et al. [40] reported that DMF induces necroptosis and elevates autophagy markers in colon cancer cells, yet autophagy inhibition did not prevent cell death, indicating a non-protective role. Consistently, our data show sustained autophagy alongside metabolic collapse and progressive cell death under DMF/ABT-199, supporting a maladaptive, cytotoxic role for autophagy in this context.

In addition to metabolic and cell death effects, DMF/ABT-199 more effectively inhibited CRC cell proliferation than either agent alone, inducing sustained G2 arrest with reduced S-phase entry. This was accompanied by downregulation of cyclins A2/B1 and CDKs 1/4/6. While DMF alone caused only a transient arrest—likely due to its short half-life [15]—the addition of ABT-199 produced a more durable blockade. Previous studies support the role of Bcl-2 inhibition in modulating cell cycle regulators. For instance, in breast and leukemia models, ABT-199 treatment led to a G0/G1 arrest accompanied by downregulation of cyclin D1 and E2F1, likely through enhanced degradation of these proteins [41]. In addition, metabolic stress, such as ATP depletion and mitochondrial dysfunction, can activate energy-sensitive checkpoint pathways that contribute to cell cycle arrest [42]. These findings suggest that DMF/ABT-199 may promote cell cycle arrest through complementary effects on cell cycle regulators and metabolic stress responses.

The observed reductions in cyclin A2, cyclin B1, and CDK1 are consistent with impaired G2/M progression, but changes in protein abundance alone do not establish the underlying checkpoint mechanism. Entry into mitosis is regulated not only by cyclin B1–CDK1 abundance but also by CDK1 phosphorylation, phosphatase activity, checkpoint kinase signaling, and the cellular metabolic state. In this context, the pronounced ATP depletion and mitochondrial dysfunction observed after combined DMF/ABT-199 treatment may contribute to activation of energy-sensitive cell cycle checkpoints. Thus, the cyclin/CDK changes identified here should be interpreted as molecular correlates that may reinforce the G2 arrest rather than as proof of a single causal pathway.

Taken together, our results show that the combination of DMF and ABT-199 exerts a multi-faceted cell death-inducing effect in CRC cells by targeting key vulnerabilities: apoptotic resistance, redox imbalance, mitochondrial dysfunction, metabolic stress, autophagy, and cell cycle progression. However, the present study was designed to characterize the mechanistic consequences of combined DMF/ABT-199 treatment at selected concentrations rather than to formally determine pharmacological synergy. A definitive assessment of additivity or synergy will require concentration-response studies using multiple concentrations of both compounds, followed by quantitative synergy modeling such as Bliss independence, Loewe additivity, ZIP, or combination-index analyses. We have therefore interpreted the current data as evidence for an enhanced combined effect rather than as formal proof of synergy. These effects go beyond those observed with either compound alone [14,15,18] and are consistent with prior findings in CTCL models [43], while providing the first evidence for such a combination-based mechanism in colorectal cancer.

From a translational perspective, the DMF/ABT-199 combination holds strong promise. Both agents are already approved, DMF for autoimmune diseases and ABT-199 for Bcl-2-driven hematologic malignancies, facilitating rapid clinical translation [24]. Their well-characterized pharmacokinetics and manageable safety profiles support evaluation in solid tumors. The DMF concentrations used in this study were selected based on our previous preclinical work in CTCL cell lines, primary malignant T cells from patients with Sézary syndrome, and CTCL mouse models, in which comparable concentrations produced robust biological effects [14,15,43]. These preclinical findings were subsequently supported by clinical data from our multicenter phase II study in relapsed or refractory cutaneous T-cell lymphoma/Sézary syndrome, demonstrating clinical activity and favorable tolerability of oral DMF treatment [18]. For ABT-199/venetoclax, the 10 µM concentration used here was chosen as a mechanistic in vitro concentration to assess whether Bcl-2 inhibition enhances DMF-induced effects in colorectal cancer cells. We acknowledge that 10 µM, corresponding to approximately 8.7 µg/mL, is above the reported mean steady-state maximum plasma concentration (Cmax) of venetoclax at the approved 400 mg once-daily dose, which is approximately 2.1 ± 1.1 µg/mL [44,45]. Thus, the ABT-199 data should be interpreted as mechanistic preclinical evidence rather than as a direct reflection of average clinical plasma exposure. Patient stratification based on Bcl-2 expression, NF-κB activity, or metabolic biomarkers could enable personalized therapy. Future in vivo studies using xenograft or organoid models should validate these findings and clarify the role of autophagy. Incorporating autophagy inhibitors or mitochondrial modulators may further enhance efficacy.

In conclusion, we identify DMF plus ABT-199 as a mechanistically distinct, preclinically effective strategy to overcome therapeutic resistance in colorectal cancer. By inducing apoptosis, mitochondrial stress, metabolic collapse, autophagy, and cell cycle arrest, this combination targets multiple survival pathways and exemplifies the potential of drug repurposing to extend the use of established agents from hematologic malignancies to solid tumors. An important limitation of the present study is that the therapeutic index of the DMF/ABT-199 combination against normal colon epithelial cells was not directly assessed. Although our previous multicenter phase II study in patients with relapsed or refractory cutaneous T-cell lymphoma/Sézary syndrome demonstrated favorable clinical tolerability of DMF, with predominantly mild adverse events and no evidence of relevant intestinal tissue damage [18], these data do not replace a formal selectivity analysis in normal colon epithelial models. Future studies should therefore compare the cytotoxic and metabolic effects of DMF, ABT-199, and their combination in colorectal cancer cells and normal colon epithelial cells, including primary colon epithelial cultures or normal colon organoids, to define tumor selectivity and potential safety margins.

## 5. Conclusions

This study demonstrates that DMF markedly sensitizes CRC cells to Bcl-2 inhibition by venetoclax through coordinated disruption of mitochondrial respiration, redox balance, and cell cycle control. The combination induces a profound energetic crisis characterized by mitochondrial hyperpolarization, ATP depletion, sustained autophagy, and durable G2 cell cycle arrest, culminating in caspase-dependent apoptosis. By simultaneously targeting apoptotic resistance and metabolic vulnerability, DMF and ABT-199 exert enhanced anti-tumor effects that exceed those of either agent alone. Given their established clinical use and favorable safety profiles, this combination represents a promising candidate for further preclinical evaluation as a repurposed therapeutic strategy in colorectal cancer.

## Data Availability

The data presented in this study are available from the corresponding author upon reasonable request.

## Supplementary Materials

The following supporting information can be downloaded at: https://www.mdpi.com/article/10.3390/life16081250/s1. Supplementary Figure S1: Effects of ABT-199 and DMF on apoptosis induction and intracellular ATP levels in HCT-15 cells. (A) Flow cytometric analysis of apoptosis by Annexin V/DAPI staining in HCT-15 cells treated with DMSO as control, 10 µM ABT-199, 50 µM DMF, or the combination of 10 µM ABT-199 and 50 µM DMF. ABT-199 induced pronounced apoptosis, whereas DMF alone had only a minor apoptotic effect. Combined treatment with DMF and ABT-199 caused only a slight additional increase in cell death compared with ABT-199 alone, with cell death remaining below 30%. Data are presented as mean ± SD from three independent experiments (*n* = 3). (B) Intracellular ATP levels were determined by luminometric measurement after treatment with DMSO, 10 µM ABT-199, 25 or 50 µM DMF, or the combination of 10 µM ABT-199 with 25 or 50 µM DMF for 24 or 48 h. ATP levels are shown relative to the DMSO-treated control and expressed as percentage decrease. Despite the comparatively moderate induction of cell death, treatment resulted in a pronounced reduction in intracellular ATP levels, exceeding 60% under the most effective treatment condition. Data are presented as mean ± SD from three independent experiments (*n* = 3).
