# Supplementary material for: Dimethyl Fumarate Enhances Venetoclax-Induced Cell Death by Inhibiting Mitochondrial Respiration and Cell Cycle Control in Colorectal Cancer
Source: Life (Basel). 2026 Jul 28;16(8):1250. doi: 10.3390/life16081250 (PMC13514823; doi:10.3390/life16081250)
Supplement: Supplementary file 1 [file life-16-01250-s001.zip › life-4473427-supplementary.pdf]

Supplementary material:

Figure S1:

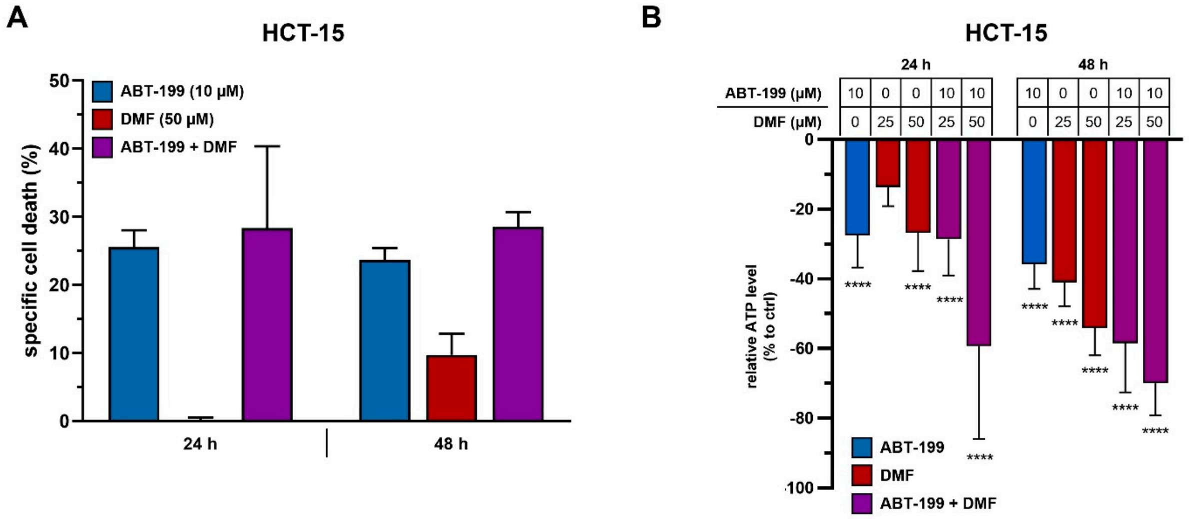

Supplementary Figure S1: Effects of ABT-199 and DMF on apoptosis induction and intracellular ATP levels in HCT-15 cells. (A) Flow cytometric analysis of apoptosis by Annexin V/DAPI staining in HCT-15 cells treated with DMSO as control, 10 µM ABT-199, 50 µM DMF, or the combination of 10 µM ABT-199 and 50 µM DMF. ABT-199 induced pronounced apoptosis, whereas DMF alone had only a minor apoptotic effect. Combined treatment with DMF and ABT-199 caused only a slight additional increase in cell death compared with ABT-199 alone, with cell death remaining below 30%. Data are presented as mean ± SD from three independent experiments (*n* = 3). (B) Intracellular ATP levels were determined by luminometric measurement after treatment with DMSO, 10 µM ABT-199, 25 or 50 µM DMF, or the combination of 10 µM ABT-199 with 25 or 50 µM DMF for 24 or 48 h. ATP levels are shown relative to the DMSO-treated control and expressed as percentage decrease. Despite the comparatively moderate induction of cell death, treatment resulted in a pronounced reduction in intracellular ATP levels, exceeding 60% under the most effective treatment condition. Data are presented as mean ± SD from three independent experiments (*n* = 3).
